# Evaluation of the Biodegradation Efficiency of Four Various Types of Plastics by *Pseudomonas aeruginosa* Isolated from the Gut Extract of Superworms

**DOI:** 10.3390/microorganisms8091341

**Published:** 2020-09-02

**Authors:** Hyun Min Lee, Hong Rae Kim, Eunbeen Jeon, Hee Cheol Yu, Sukkyoo Lee, Jiaojie Li, Dae-Hwan Kim

**Affiliations:** 1 School of Undergraduate Studies, College of Transdisciplinary Studies, Daegu Gyeongbuk Institute of Science and Technology, Daegu 42988, Korea; gusals522@dgist.ac.kr (H.M.L.); chandelle98@dgist.ac.kr (H.R.K.); eunbini98@dgist.ac.kr (E.J.); kirby@dgist.ac.kr (H.C.Y.); 2 Department of Brain and Cognitive Sciences, Daegu Gyeongbuk Institute of Science and Technology, Daegu 42988, Korea; slee2012@dgist.ac.kr; 3 Department of Chemistry, Gwangju Institute of Science and Technology, Gwangju 61005, Korea

**Keywords:** *Pseudomonas aeruginosa*, plastic biodegradation, gut bacteria, superworm, enzyme

## Abstract

Plastic waste worldwide is becoming a serious pollution problem for the planet. Various physical and chemical methods have been tested in attempts to remove plastic dumps. However, these have usually resulted in secondary pollution issues. Recently, the biodegradation of plastic by fungal and bacterial strains has been spotlighted as a promising solution to remove plastic wastes without generating secondary pollution. We have previously reported that a *Pseudomonas aeruginosa* strain isolated from the gut of a superworm is capable of biodegrading polystyrene (PS) and polyphenylene sulfide (PPS). Herein, we demonstrate the extraordinary biodegradative power of *P. aeruginosa* in efficiently depolymerizing four different types of plastics: PS, PPS, polyethylene (PE) and polypropylene (PP). We further compared biodegradation rates for these four plastic types and found that PE was biodegraded fastest, whereas the biodegradation of PP was the slowest. Moreover, the growth rates of *P. aeruginosa* were not always proportional to biodegradation rates, suggesting that the rate of bacterial growth could be influenced by the composition and properties of intermediate molecules produced during plastic biodegradation, and these may supply useful cellular precursors and energy. In conclusion, an initial screening system to select the most suitable bacterial strain to biodegrade certain types of plastic is particularly important and may be necessary to solve plastic waste problems both presently and in the future.

## 1. Introduction

Plastic products have various advantages, such as being light-weight and flexible, possessing high physical strength, and being long-lasting compared to natural materials [1]. Plastic has been used in many industries and in daily life due to its low-cost and easy production, which results in tremendous amounts of plastic waste across the planet every day [2]. Generally, plastic degradation proceeds extremely slowly under natural conditions. As a consequence, plastic waste accumulates everywhere, including in the sea [3], and has become an extremely serious issue globally. Although many physical and chemical techniques to remove plastic wastes have been developed, such methods often lead to secondary pollution problems [4]. For example, microplastics produced from landfilled plastic eventually flow to oceans via erosion, damaging marine organisms and causing the derangement of aquatic ecosystems generally. The incineration of plastic wastes causes serious air pollution and threatens human health [5]. Therefore, harmless techniques to remove plastic wastes are urgently required. Plastic biodegradation trials using various microorganisms have been intensively studied. 

Recently, several plastic-ingesting worms capable of removing plastic wastes have been reported, including waxworms (*Galleria mellonella* L.), mealworms (*Tenebrio molitor* L.) and superworms (*Zophobas atratus* L.), which can depolymerize polyethylene (PE) and polystyrene (PS) upon ingestion [6,7,8,9,10,11,12,13,14]. Further studies have shown that treatment with antibiotic compounds to remove gut bacteria within the worms significantly reduces the biodegradation efficiency with respect to ingested PE and PS, suggesting that gut bacteria participate in plastic biodegradation in these worms [9,10,15]. Several gut bacterial species isolated from worms have been verified to be able to mediate plastic biodegradation directly. *Enterobacter asburiae* YT1 and *Bacillus* sp. YP1 isolated from waxworms can depolymerize PE in vitro [16]. *Exiguobacterium* sp. strain YT2 isolated from mealworms can biodegrade PS successfully [15]. Newly-isolated *Acinetobacter* sp. AnTc-1 from a larva of *Tribolium castaneunm* can mediate the biodegradation of PS [17]. Thus, the gut bacteria of these worms play an essential role in the plastic biodegradation process. 

Although the pathogenic *Pseudomonas* sp. can cause infections after surgery in the human body, such as in the blood and the lung, various *Pseudomonas* sp. strains isolated from several different places, including soil, plastic trash and even the deep sea, have been intensively studied in relation to plastic biodegradation for a long time. *Pseudomonas* sp. can depolymerize several plastic types, including polyvinyl chloride, polyurethane, polyvinyl alcohol, PE and PS [18]. *P. aeruginosa*, isolated from the gut of superworms, was shown to be able to degrade PE, PS and polyphenylene sulfide (PPS) in an earlier work from our group [8,19]. We have further identified and verified that serine hydrolase (SH), a candidate enzyme, participates in the plastic biodegradation process, via RT-qPCR analyses and enzyme inhibitor treatment assays [8]. Besides *Pseudomonas* sp., several other gut bacteria in worms have also been reported to participate in the biodegradation process of ingested plastics [15,16,17]. Although, single bacterial species can degrade several plastic types, the biodegradation efficiencies toward each different type of plastic vary enormously [18,19]. Herein, we have compared the biodegradation efficiencies of a *P. aeruginosa strain* toward four different types of plastic: PE, PS, PPS and polypropylene (PP). This study can be applied to select a suitable bacterial species to degrade certain types of target plastic more efficiently in the future.

## 2. Materials and Methods

### 2.1. Isolation of P. aeruginosa from the Gut Extract of Superworms

Styrofoam PS (99.5% purity, Myung-IL FOAMTEC, Daegu, South Korea) was supplied as the sole carbon food source to enrich the PS-degrading bacteria in guts of these superworms. The isolation of gut bacteria from fourth instar larvae of superworms was following our previous protocols [8,19]. The gut was extracted from larvae anesthetized by immersing in 70% ethanol and then washed with saline water (SW). To isolate gut bacteria, the supernatant layer was carefully transferred to a flask containing liquid carbon-free basal medium (LCFBM) after centrifugation of the grounded guts and the mixed solution was cultured in a shaking incubator at 25 °C with a speed of 180 rpm for 60 days with PS films (99.9% purity, Goodfellow, Huntingdon, UK). The diluted cultured gut solution (2 mL) with LCFBM solution (1:100) was spread onto a solid nutrient medium (BD Difco, Franklin Lakes, NJ, USA). After 2–3 days, each colony grown on the solid nutrient medium was identified by 16S rRNA gene sequence analysis. NCBI BLAST program was used for the identification of bacterial species. 

### 2.2. Examining Growth of P. aeruginosa on PS Films

Bacterial pellets were collected from 5 mL liquid bacteria culture medium by centrifuging for 5 min at 4000 rpm. After removing the nutrient medium and washing with liquid LCFBM solution three times, the bacteria pellets were resuspended in 1 mL LCFBM liquid media and spread onto LCFBM plates. Sterilized PS films were then placed on LCFBM plates. After drying plates at room temperature under sterile conditions, plates were sealed with parafilm and incubated at 25 °C for 40 days. Bacterial colonies were then examined. 

### 2.3. Scanning Electron Microscopy (SEM) Analysis for PS Biodegradation

SEM (SU8230, Hitachi, Tokyo, Japan) was used to verify the proliferation and adherence of *P. aeruginosa* on the surface of PS beads and PS degradation mediated by the bacteria. To examine changes on the PS surface caused by *P. aeruginosa*-mediated biodegradation, PS beads from LCFBM liquid culture media were dried. Adhered bacteria were removed by soaking with 2% sodium dodecyl sulfate (SDS) for 4 h. PS beads were then washed with DW three times to remove SDS. The PS beads were then dried, fixed on copper tapes, and subjected to platinum coating using an ion sputter for 15 s at 15 mA. SEM was used to examine the surfaces of PS beads. All images were obtained at an acceleration voltage of 1.6 kV.

### 2.4. Analysis of Elemental Compositions on the Surface of Plastic

X-ray energy dispersive spectroscopy with a module attached to the SEM was used to determine changes of elemental compositions on the surface of plastic beads during *P. aeruginosa-mediated* plastic biodegradation. Areas exhibiting proliferated *P. aeruginosa* were observed to show changes in elemental compositions. Plastics without bacteria were used as control groups to analyze differences compared to the test groups.

### 2.5. Analysis of Fourier-Transform Infrared Spectroscopy (FT-IR) for PP Beads

To determine changes to the chemical structure of biodegraded PP caused by the *P. aeruginosa*, FT-IR (Nicolet Continuum, Thermo Fisher Scientific, Waltham, MA, USA) was used. PP beads retained by a 70 μm sieve were dried at 60 °C for 24 h after removing bacteria with 2% SDS and washing twice with deionized sterile water. The small hydrophilic PP particles collected after passing through the sieve were used to obtain the FT-IR spectra. The smart single-bounce attenuated total reflectance with a 4400–400 cm^−1^ range diamond tip was used.

### 2.6. Measurement of Mass Reduction of Biodegraded Plastic beads

To compare the *P. aeruginosa-mediated* biodegradation efficiencies for four different types of plastics (PE, PS, PPS and PP), 1 g of each type of plastic bead was used to measure mass reduction quantitively. Biodegradation reactions were performed in liquid LCFBM for 15 days at 25 °C with shaking at 180 rpm. Biodegraded plastic beads were collected through a 70 μm sieve and a 0.45 μm filter. The bacteria attached to beads were removed by 2% SDS soaking followed by DW rinsing three times. After drying at 60 °C for 24 h, mass reductions of different types of plastic beads were measured independently in triplicate. Under the same conditions, the liquid LCFBM containing each type of plastic bead but with no bacteria was tested as controls.

### 2.7. Comparison of Growth Rates of P. aeruginosa Via Optical Density (OD) Measurement and Colony Counting

To compare the *P. aeruginosa-mediated* biodegradation efficiencies for four different types of plastics (PE, PS, PPS and PP), 1 × 10^8^ *P. aeruginosa* were inoculated in 25 mL liquid LCFBM media containing 2 g of one type of plastic bead. During an 8-day period, the optical density (OD) of 1 mL liquid LCFBM media containing *P. aeruginosa* was measured at 600 nm to evaluate bacterial growth rate every day. Colony counting was also performed. After serial dilution of 1 mL of liquid LCFBM medium containing bacteria and different types of plastics, the liquid medium was spread onto a nutrient solid media plate and the number of growing colonies was counted after 2–3 days. 

## 3. Results 

### 3.1. Survival of P. aeruginosa Supplied with PS As the Sole Carbon Source

**Figure 1:**
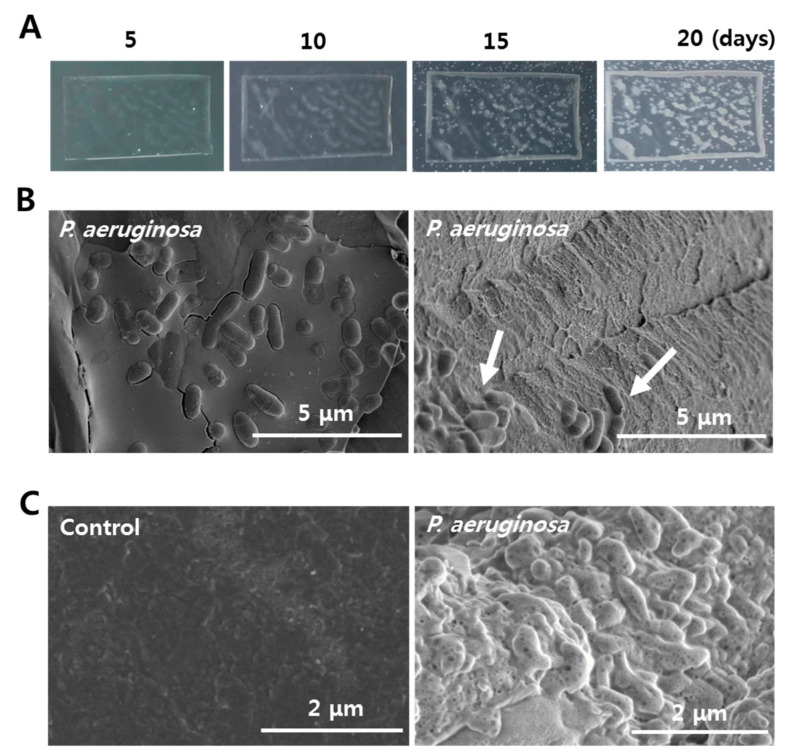
Isolation of polystyrene (PS)-degrading *P. aeruginosa* from the gut extract of PS-ingesting superworms. (**A**) Growth of *P. aeruginosa* on PS films during a 20-day period. White color on the middle and sides of the PS film shows growth of *P. aeruginosa.* (**B**) Biodegradation of PS beads by *P. aeruginosa*. Before (left) and after SDS treatment (right) are shown. The remaining *P. aeruginosa* were still attached onto the surfaces of PS beads (indicated by white arrow). (**C**) Surface changes on PS beads during biodegradation by *P. aeruginosa* (right). PS beads without bacteria were used as controls (left).

Two different species of bacteria were observed on PS films when the enriched gut bacteria of superworms were incubated in PS-added LCFBM media. Bacterial colonies started to appear on the surfaces of PS films after four days. One major type of bacteria grew in a large-sized oval form with a translucent gray color within the PS film. The growing area of the gray-colored colony was much larger than that of the other white colored bacteria (Figure 1A). After 10 days, bacteria with a translucent gray color were enriched on the square sides of PS films. They populated over 50% of the surface area, suggesting that their growing speed was much faster than that of the white colony bacteria. After 15 days, the number of white colonies on the PS film and LCFBM agar plate beyond the PS film was significantly increased, unlike bacteria with a translucent gray color (Figure 1A). Based on 16S rRNA sequencing analysis, the bacterial cells of the translucent gray-colored colony on the surface of PS film were identified as *P. aeruginosa* (GenBank:MT045992.1). Therefore, *P. aeruginosa* both survived and thrived under a nutrient-deficient environment with PS as the sole carbon source.

In the incubation with PS beads in LCFBM solution, numerous *P. aeruginosa* were attached on the surfaces of the PS beads and the amplification of bacterial populations via cell division was also observed (Figure 1B, left). After removing most attached bacteria with SDS treatment, PS beads still had a few remaining bacteria adhering, and showed a strong surface corrosion after biodegradation in liquid LCFBM media for 20 days (Figure 1B, right), unlike the control group without bacteria (Figure 1C). 

### 3.2. Biodegradation of PS Beads by P. Aeruginosa 

**Figure 2:**
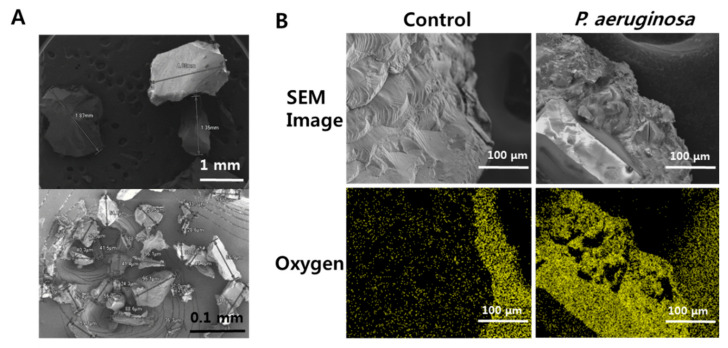
SEM analyses of PS beads biodegraded by *P. aeruginosa.* (**A**) Comparison of size reduction between PS beads biodegraded by *P. aeruginosa* (lower) and PS beads without bacteria (upper). (**B**) Detection of oxygen insertion on surfaces of PS beads. The yellowish-green color represents oxygen atoms in control without bacteria (left) and on beads biodegraded by *P. aeruginosa* (right).

We observed that the diameters of many PS beads were significantly reduced after they were incubated with *P. aeruginosa* in liquid media compared to PS beads in the control group (Figure 2A). The reduced size of PS beads demonstrated that *P. aeruginosa*-mediated biodegradation was actively progressing. Elemental composition analysis showed strong oxygen signals on the surfaces of PS beads incubated with *P. aeruginosa*, but not on the surfaces of PS beads in the control group. (Figure 2B). Oxygen insertion on the plastic surface indicated that oxidation had occurred during the biodegradation process. 

### 3.3. Comparison of Biodegradation Efficiencies for PE, PS, PPS and PP by P. aeruginosa 

**Figure 3:**
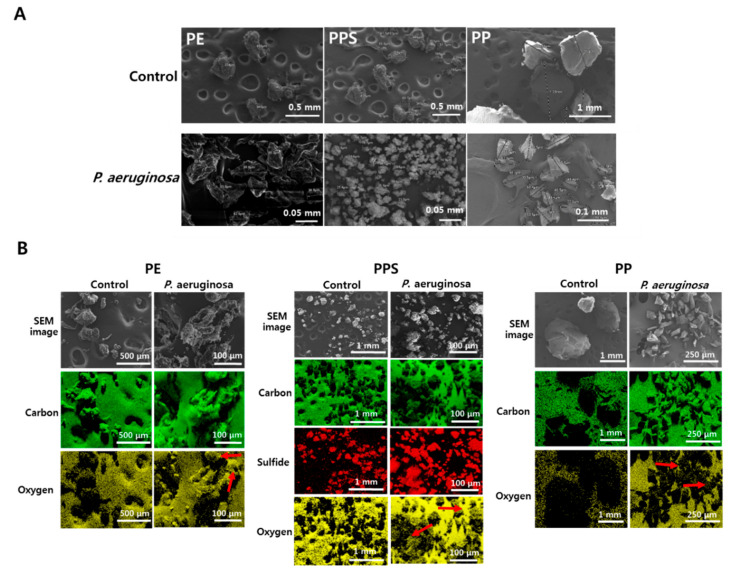
Scanning electron microscope (SEM) and elemental composition analyses of polyethylene (PE), polyphenylene sulfide (PPS) and polypropylene (PP) beads incubated with *P. aeruginosa.* (**A**) Comparison of size reductions for PE (left), PPS (middle) and PP (right) beads biodegraded by *P. aeruginosa* (lower) and control beads without bacteria (upper). (**B**) Detection of elemental composition on surfaces of three types of plastic beads, PE (left), PPS (middle) and PP (right). Green, yellow and red colors represent carbon, oxygen and sulfur (PPS) atoms. Unlike control without *P. aeruginosa* (left column), oxygen was strongly detected on the surface of plastic beads biodegraded by *P. aeruginosa* (red arrows in right column).

In the biodegradation test of other plastic types, the diameters of the PE and PPS beads were both significantly reduced after these beads were incubated with *P. aeruginosa* in the liquid LCFBM media (Figure 3A). Unlike the other two types of plastics, the diameters of most PP beads were not reduced, suggesting that PP was not properly degraded by *P. aeruginosa* (Figure 3A). Elemental composition analyses showed strong oxygen signals on the surfaces of both the PE and PPS beads, confirming that similar oxygen insertion occurred during the biodegradation of these two types of plastics as seen for PS (Figure 3B). In contrast, much weaker oxygen signals were detected on the surfaces of PP beads, indicating that the *P. aeruginosa*-mediated biodegradation of PP did not proceed as actively (Figure 3B).

**Figure 4:**
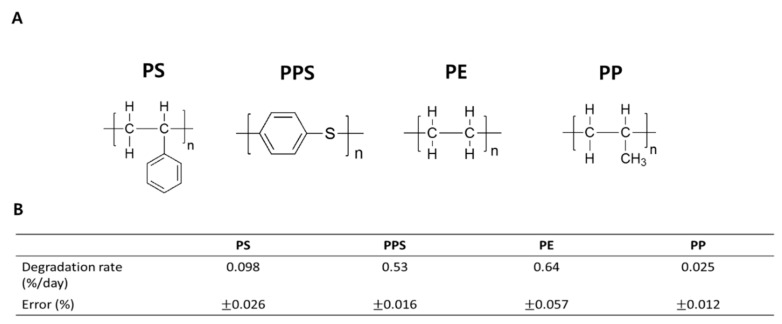
Comparison of biodegradation rate of PS, PPS, PE and PP by *P. aeruginosa*. (**A**) Chemical structures of the four different types of plastic. (**B**) Biodegradation rates for all plastics. Biodegradation rate of each plastic type per day (%) was calculated by dividing the total weight change by the number of incubation days.

To compare biodegradation efficiencies for four different types of plastic (PE, PS, PPS and PP) by *P. aeruginosa*, we quantitively measured weight reductions for each type of plastic after a 15-day biodegradation process in a liquid LCFBM solution (Figure 4A). Among these four plastics, PE had an average weight loss of 0.64% per day from the total, which was the highest (Figure 4B). The biodegradation speed of PPS was the second fastest, showing a weight loss of 0.53% per day (Figure 4B). The weight reductions of PS and PP were much slower (at 0.098% and 0.025% per day, respectively), indicating reduced biodegradation efficiencies for PS and PP by *P. aeruginosa* (Figure 4B). Therefore, *P. aeruginosa* demonstrated very different biodegrading capabilities for the four plastics.

### 3.4. FT-IR Analyses of PP Biodegradation by P. aeruginosa 

**Figure 5:**
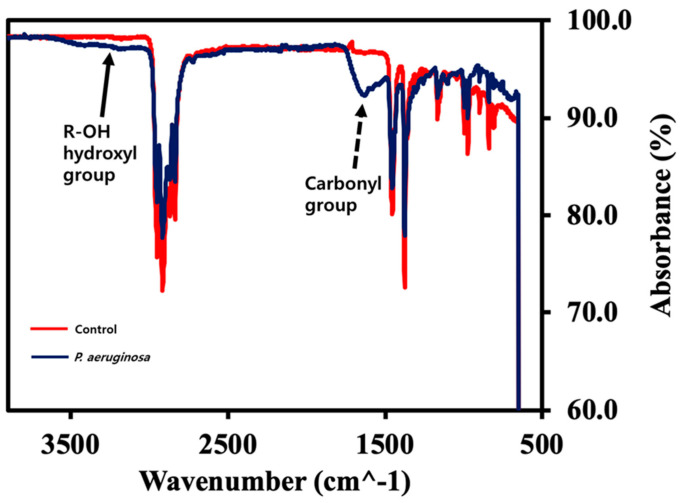
FT-IR analysis of chemical structural changes during PP biodegradation. FT-IR spectra of PP beads biodegraded by *P. aeruginosa* (blue line) versus control PP beads (red line). Solid line arrow, hydroxyl group (-O-H); Dotted line arrow, carbonyl group (-C=O).

Both elemental composition analyses and weight reduction measurements showed that the *P. aeruginosa*-mediated biodegradation of PP did not actively proceed. To check the biodegradation of PP by *P. aeruginosa*, we further conducted FT-IR to analyze the chemical structural changes of PP beads. Compared to PP in the control, representative absorptions of carbonyl (−C=O) at 1715 cm^−1^ and hydroxyl (−O–H) at 3300–3600 cm^−1^ identified that *P. aeruginosa*-mediated PP biodegradation had occurred (Figure 5), however the efficiency for PP was much lower than that for PE, PS or PPS (Figure 4B). 

### 3.5. Effects of Plastic Types on the Growth of P. aeruginosa 

**Figure 6:**
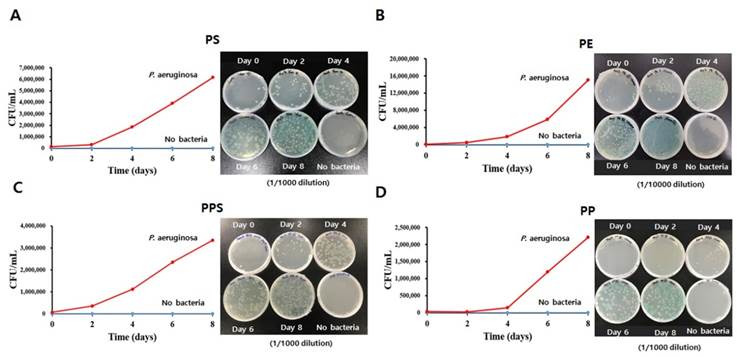
Comparison of growth rate of *Pseudomonas aeruginosa* when supplied with PS, PE, PPS or PP as the sole carbon source. Graph (left) and plates (right) of counted colony-forming units (CFU) of *P. aeruginosa* in liquid LCFBM supplied with 1 g of PS (**A**), PE (**B**), PPS (**C**) or PP (**D**) for eight days.

Bacteria have to utilize plastic to produce energy for their survival and amplification when it is the sole available carbon source. Thus, the biodegradation efficiencies of different types of plastics could affect the growth rates of *P. aeruginosa* and change their population characteristics [19]. To compare the effects of different plastic types on the growth of *P. aeruginosa*, we measured changes in the bacteria number in a liquid LCFBM media supplied with different plastic. After eight days, the greatest number of *P. aeruginosa* were observed in the PE-added LCFBM media (16 × 10^6^), consistent with its highest biodegradation rate among the four plastics (Figure 6B). The number of *P. aeruginosa* was 6.3 × 10^6^ in PS-added LCFBM media (Figure 6A). In contrast, the bacteria numbers were relatively lower under PPS- and PP-replete conditions (3.6 × 10^6^ and 2.5 × 10^6^ of *P. aeruginosa*, respectively) (Figure 6C and 6D). Especially in PP-media, the bacterial population did not show any growth during the first four days (Figure 6D). Interestingly, the *P. aeruginosa-*mediated biodegradation rate for PPS was much faster than that for PS (Figure 4B), although the number of amplified bacteria in the PPS-supplied condition was approximately half of that in the PS-supplied condition (Figure 6). This suggests that other factors besides simply the plastic substrate might be involved in bacterial amplification and biodegradation efficiency. 

## 4. Discussion 

Various bacterial species residing in the gut of insect larva participate in the biodegradation of ingested plastics [8,14,15,16]. However, due to inevitable environmental differences, including oxygen level, pH and concentrations of various ions between the gut environment of the insect larva and in vitro enrichment systems, the most efficient plastic-biodegrading bacteria in the gut are not always successfully isolated or match bacteria that thrive using in vitro enrichment methods. For example, the most effective PS-biodegrading anaerobic bacteria strains in the gut of insect larva cannot survive in an in vitro environment supplied with oxygen. However, *P. aeruginosa* classified as aerobes or facultative anaerobes can survive and grow in both aerobic and anaerobic conditions [20,21]. In addition, *P. aeruginosa* can survive in various places, including soil, wood, water, dumpsites, the deep sea and the guts of larvae of superworms, suggesting that it is highly adaptive [22,23,24]. It not only degrades plastic in the guts of larva, but also in our in vitro systems with plastic-added LCFBM media (Figure 4B) [8,14].

In this study, we demonstrated the biodegradation of four different types of plastics, PE, PS, PPS and PP, by *P. aeruginosa*, and further compared the biodegradation efficiencies for each. The *P. aeruginosa* used in this study was prepared and enriched by isolating the gut extract from PS-ingested superworms, followed by culturing in PS-added liquid LCFBM media for 60 days [8]. Our current results as well as our previous studies have shown that a single bacteria strain can depolymerize several different types of plastics, although with different efficiencies [19] (Figure 4B). The *P. aeruginosa*-mediated PE biodegradation efficiency in this current work was much faster than for any of the other plastic types tested (PS, PPS or PP) (Figure 4B). 

The efficiency of plastic biodegradation is highly determined by the amount of expressed enzyme and the catalytic ability of the secreted enzymes [25,26,27], and through hydrolysis can change the surface property from hydrophobic to hydrophilic, thus reducing the plastic’s mechanical strength [8,28,29,30]. Recently, several enzymes that participate in plastic biodegradation have been reported, including serine hydrolase (SH) secreted from *P. aeruginosa*, as identified by our group [8,29,31,32,33]. Thus, the more efficient biodegradation of PE might be because depolymerases secreted from *P. aeruginosa* can mediate the depolymerization of PE better than the other kinds of plastics due to the strong binding affinity of the enzyme for PE, or because certain specific PE-degrading enzymes are more highly expressed than others. Our previous studies have shown that oxidation is a crucial step that initiates the entire bacteria-mediated biodegradation process and progresses the subsequent bio-deterioration, bio-fragmentation, assimilation and mineralization [8,30,34,35]. Thus, the observed strong oxygen insertion signal on the PE surface demonstrated that oxidation was more actively advanced on PE compared to PPS or PP. This might also have contributed to the greater biodegradation efficiency for PE (Figure 3B). 

When plastic is supplied as the sole carbon source, bacteria have to obtain energy from plastic biodegradative byproducts so as to generate cellular components and maintain cellular activity and reproduction via cell division [36,37]. Fragments produced from PE depolymerization could be easily converted to fatty acids via enzyme-mediated oxidation steps, and then subsequently converted to acetyl-CoA for energy production via beta-oxidation and the TCA cycle [38]. The supply of sufficient energy and other nutrients resulting from rapid PE depolymerization allowed steep population increases of *P. aeruginosa* (Figure 6B). A previous study showed that pyrolysis of PE by *P. aeruginosa* PAO-1 can produce polyhydroxylalkanoate (PHA) [39]. Thus, PE biodegradation is an effective process by which *P. aeruginosa* can obtain energy under nutrient-limiting conditions. In contrast, the growth rate of *P. aeruginosa* was very slow when PP, which has a similar carbon skeletal structure, was supplied as the sole carbon source (Figure 6D). This result might be due to the low gene expression level or even the absence of certain PP-degrading enzymes in *P. aeruginosa*, meaning that defective enzyme functions could then reduce the PP biodegradation rate. It has been reported that *Yarrowia lipolytica* 78-003, a bacterial strain, can convert PP to fatty acids and then to the energy source PHA [40]. Therefore, insufficient raw carbon fragments resulting from the extremely slow PP biodegradation rate and/or the defective enzyme function for converting PP to the corresponding energy source PHA by *P. aeruginosa* might have further limited the increase of the bacterial population (Figure 6D). 

The chemical structures of both PS and PPS have an aromatic ring in common (Figure 4A). A greater number of enzyme-mediated reaction steps are required to open the aromatic ring to produce acetyl-Co A, or to succinate for the TCA cycle and PHA for energy production [41]. Thus, rather more complicated enzyme systems could be one of the reasons for their slowed biodegradation rates compared with PE (Figure 4B). The relatively weaker -C-S bond in PPS might have accelerated its biodegradation rate compared to PS. Meanwhile, the more limiting energy supply as a result of the less efficient biodegradation reduced bacterial growth in both plastic types (Figure 6A,C). Interestingly, the population of *P. aeruginosa* increased about twice as much in the presence of PS compared to PPS (Figure 6A,C), even though the PS biodegradation rate was much slower than the PPS rate (Figure 4B). Earlier work from our group has shown that benzenesulfonic acid with a strong acidity is most likely to be formed as an intermediate molecule during the PPS biodegradation pathway [19]. A low pH condition in the bacterial cytoplasm during PPS depolymerization might have impaired the functions of enzymes involved in energy production and cell division, thus significantly interfering with bacteria growth (Figure 6C) [42]. Therefore, the different chemical structures of PE, PS, PPS and PP required the collaboration of different combinations of enzymes secreted by *P. aeruginosa* in order to mediate their biodegradation, thus leading to their different biodegradation efficiencies. Theoretically, a faster biodegradation rate could provide more carbon fragments to produce cellular components and energy for bacterial amplification. However, convenience and efficiency in converting these raw materials into useful products might also play an important role in bacteria growth. 

Our current study demonstrated the extraordinary biodegradative capabilities of *P. aeruginosa* isolated from the guts of superworms for four different types of plastics (PE, PS, PPS and PP). However, the biodegradation efficiency varied from one plastic type to another, with biodegradation of PE occurring fastest. We believe that the biodegradation rate, and the ease and efficiency with which those raw materials are converted to useful cellular components and energy, together determine bacteria growth. The selectivity resulting from different biodegradation rates for various plastic types by single bacteria strains could be utilized to conduct the fine purification in the late stages in the plastic recycling industry in future.
